# Low-Frequency STN-DBS for Standing and Gait Initiation in Parkinson’s Disease: A Double-Blinded Randomised Cross-Over Feasibility Trial

**DOI:** 10.1177/15459683261448483

**Published:** 2026-06-04

**Authors:** Zachary J. Conway, Peter A. Silburn, Thushara Perera, Karen O’Maley, Liam Johnson, Michael H. Cole

**Affiliations:** 1 School of Allied Health, Australian Catholic University, Brisbane, QLD, Australia; 2 School of Behavioural and Health Sciences, Australian Catholic University, Brisbane, QLD, Australia; 3 Neurosciences Queensland, Brisbane, Australia; 4 The Bionics Institute, East Melbourne, VIC, Australia; 5 Medical Bionics Department, The University of Melbourne, VIC, Australia; 6 School of Behavioural and Health Sciences, Australian Catholic University, Melbourne, QLD, Australia; 7 Development and Disability Over the Lifespan, Healthy Brain and Mind Research Centre, Australian Catholic University, Brisbane, QLD, Australia

**Keywords:** gait, fall, Parkinson’s disease

## Abstract

**Background:**

Following high-frequency (>100 Hz) deep brain stimulation of the subthalamic nucleus (STN-DBS), some people with Parkinson’s disease (PD) report poorer balance which may contribute to an increased falls risk. However, low-frequency (60 Hz) STN-DBS may improve balance. To better understand the effect of low-frequency STN-DBS, this double-blind randomised crossover study evaluated the acute effects of this therapy on objective measures of balance during standing and gait initiation in people with PD.

**Methods:**

Sixteen post-operative PD patients completed standing, walking and clinical assessments while off medication and receiving high- and low-frequency STN-DBS therapy. Changes in balance were measured using a force plate to provide insight into the effect of stimulation strategy on balance during standing and gait initiation.

**Results:**

Linear mixed model analyses indicated that balance during both standing and the locomotion phase of gait initiation was improved with low-frequency STN-DBS, while no difference in the postural phase of gait initiation. These effects were independent of electrode location and total electrical energy delivered. No differences were noted between stimulation conditions for clinical measures of mobility, motor symptom severity, or gait retropulsion.

**Conclusions:**

This study provides evidence for the acute benefits of low-frequency stimulation for improving objective measures of balance during standing and gait initiation in STN-DBS PD patients, independent of DBS electrode contact location. However, the benefits of this alternate therapy may be reduced for patients who experienced significant pre-operative tremor, as low frequency was less effective for managing tremor.

**Trial Registry:**

Australian New Zealand

**Clinical Trials Registry:**

URL: www.anzctr.org.au.

**Registration number:**

ACTRN12618000944235.

## Introduction

Deep brain stimulation (DBS) of the subthalamic nucleus (STN-DBS) has become a common procedure for improving symptoms of Parkinson’s disease (PD), such as resting tremor and limb stiffness, that are typically refractory to pharmacological treatments.^1,2^ Post-operatively, people typically experience reduced symptom severity, fewer dyskinesias, and an improved quality of life.^3^ However, postural instability, a symptom strongly associated with falling in people with PD,^4^ is reported to decline following STN-DBS.^5^ The deterioration of balance following STN-DBS is considered to be a contributing factor to the increased falls rate reported for patients who are more than 1 year post-surgery.^6^

Balance is commonly evaluated by measuring the centre of pressure (COP) on a force plate to provide insight into the movements of the individual’s centre of mass (COM) and, hence, their postural sway. Assessments have traditionally focussed on balance during static (ie, standing) or steady-state dynamic (ie, walking) tasks, with a growing number of studies investigating the transition phase between these static and dynamic states (ie, gait initiation).^7-9^ Gait initiation includes 2 distinct postural and locomotion phases.^10^ During the postural phase, there is a shift in COM towards the stance leg which results in a concomitant and proportional change in position of the COP. During the locomotion phase of gait initiation, the COM is projected forward in the direction of travel, which is reflected by a simultaneous change in the trajectory of the COP.^11^

Despite the significant improvements reported for symptoms of limb tremor and rigidity, people with PD continue to experience declines in gait initiation following STN-DBS surgery.^12^ There is inconclusive evidence for the effects of alternate stimulation parameters (eg, voltage amplitude or stimulation frequency) on the post-operative management of balance.^13^ Specifically, low-frequency stimulation (60-80 Hz) has been shown to both improve^14,15^ and have no effect on clinical measures of balance when compared to high-frequency SNT-DBS.^16-18^ Though most research has focussed on the effect of low-frequency stimulation on steady state walking^13^; there is a need for further research to assess the potential benefits of alternate stimulation parameters, such as low-frequency STN-DBS for improving balance during standing and gait initiation. This study employed a double-blind randomised crossover design to evaluate the effect of low-frequency STN-DBS on objective measures of balance during standing and gait initiation in people with PD. It was hypothesised that low-frequency stimulation would significantly improve balance compared to the usual high-frequency stimulation setting.

## Methods

### Participants

Participants were recruited at random from a private neurology clinic and local support groups via a letter of invitation that outlined the study’s requirements and the potential benefits and risks of participation. People were accepted into the study if they were; clinically-diagnosed with idiopathic PD; aged between 50 and 75 years; had undergone bilateral STN-DBS surgery no less than 12 months earlier; independently living within the community; able to stand and ambulate without assistance; free of any significant musculoskeletal or medical conditions (other than PD); were not taking any medications that would adversely affect their balance or mobility; and free of any signs of dementia (Standardised Mini-Mental State Examination score >25).^19^ This study was approved by the Australian Catholic University’s Human Research Ethics Committee (2017-155H) and participants provided written informed consent prior to participation. Given the lack of reported data concerning posturographic outcomes with low-frequency STN-DBS, the study sample size was estimated based on posturographic data collected while investigating a similar patient group with and without high-frequency stimulation.^20^ It was determined that a minimum of 11 participants were required to detect differences between high- and low-frequency stimulation with respect to the COP outcomes (Effect size ≥0.49, Power = 0.8, *P* = .05).

The locations of DBS electrodes were identified by merging the postoperative CT scans with the preoperative magnetic resonance imaging data using three-dimensional (3D) Slicer v4.11.^21^ Images were aligned along the anterior and posterior commissures to normalise brain orientation using acpcdetect v2.0 (NeuroImaging Tools & Resources Collaboratory, https://www.nitrc.org). The 3D coordinates for the ideal neurosurgical target within each STN^22^ were determined separately for each hemisphere of the brain by an experienced Neurologist. These data were subsequently used to calculate the distance (in millimetres) between the midpoint of each electrode and the ideal target. The difference between the ideal and actual location of the active electrode was expressed in the form of *X* (negative = more medial), *Y* (negative = more posterior), and *Z* (negative = more inferior) distances, which were combined to provide a Euclidean distance. All distance calculations were performed automatically using a custom script written in Python v3.7 (Python Software Foundation).

### STN-DBS Interventions

Following overnight withdrawal of antiparkinsonian medications (≥12 hours), participants attended a single testing session to complete measures of balance during both high- and low-frequency STN-DBS. Testing was completed within a dedicated research space at their usual neurology clinic. On arrival, a nurse specialised in the post-operative management of STN-DBS patients and who was blinded to the patients’ performances during the testing battery determined the electrode impedance of the DBS device and calculated the total electrical energy delivered (TEED) for the participants’ chronic (high-frequency) stimulation settings by determining the product of the voltage amplitude, stimulation frequency, pulse width, and biological impedance.^23^ The order of the high- and low-frequency stimulation conditions were applied was randomised using a computer-generated randomisation sequence by a team member who had no direct involvement in data collection or analysis. Of the 16 participants recruited for this study, 10 were randomly assigned to receive high-frequency stimulation (HFS) first, while 6 received low-frequency stimulation (LFS) first. Using a randomised one-to-one allocation ratio, the DBS nurse programmed the STN-DBS electrodes to 1 of 2 therapeutic conditions; (i) high-frequency; or (ii) low-frequency stimulation. Specifically, the high-frequency condition involved the STN-DBS electrodes being bilaterally active with the high-frequency stimulation (>100 Hz) that the patients were receiving as part of their usual chronic therapy. In contrast, low-frequency stimulation involved the STN-DBS electrodes being bilaterally set to a lower frequency (60 Hz) with the voltage increased to maintain the TEED at a level consistent with the patient’s high-frequency (chronic) stimulation setting. To limit the risk of any carry-over effects between the high-frequency and low-frequency conditions (or vice versa), a 1-hour wash-in period was enforced between testing conditions.^24^ To limit the risk of bias, only the DBS nurse responsible for adjusting the stimulation parameters was aware of the STN-DBS settings for each condition; hence, both the participant and the researchers administering the assessments were blinded to the stimulation state.

### Procedures

Participants were asked to complete a series of questionnaires (Table 1) to acquire their medical history, medication use, and balance confidence. Furthermore, during each therapeutic condition, symptom severity was assessed by a trained movement scientist using part 3 (motor sub-section) of the Movement Disorders Society-Sponsored Revision of the Unified Parkinson’s Disease Rating Scale (MDS-UPDRS III). The total score and item 12 (retropulsion test) of the sub-section were reported, with higher scores for these outcomes representing greater symptom severity and/or poorer balance, respectively. Following the clinical assessment, participants were asked to complete two 30-second standing balance trials on a portable force plate (Advanced Mechanical Technology Inc., USA). During these trials, participants stood barefoot with their hands at rest by their sides, their feet parallel at 10 cm apart, and their eyes open. Participants were asked to focus their gaze on a point situated 10 m directly in front of them and to refrain from talking, unless necessary. For any trials where the participant was unable to stand quietly (eg, they spoke, coughed, and sneezed), the task was repeated. Following this, participants were asked to complete 2 barefoot walking trials along a 14-m long, level, walkway at a self-selected comfortable pace starting from a standing position on the force plate. During both the standing and walking tasks, COP data were captured by the force plate at 200 Hz.

**Table 1.** Demographic Information, Disease-specific Characteristics, Clinical Measures, Surgical Information, and Stimulation Parameters for the STN-DBS PD Participants.

Participant Information	HFS (n = 16)	LFS (n = 12)
Demographics		
Gender (male)^a^	12.0 (75.0%)	—
Age (years)	69.9 (7.5)	—
Height (m)	1.72 (0.1)	—
Mass (kg)	80.1 (14.6)	—
Falls history and fear of falls		
Retrospective faller^a^	9.0 (56.25%)	—
ABC-6	49.0% (26.1%)	—
Neurological examination		
Disease duration (years)	12.4 (6.6)	—
MDS-UPDRS-III	33.2 (10.0)	32.7 (10.9)
Freezers^a^	5.0 (31.3%)	—
New freezing of Gait Questionnaire	19.0 (5.5)	—
PDQ-8	27.7 (14.9)	—
No anti-parkinsonian medications^a^	7.0 (43.8%)	—
Levodopa dose (mg/day)	297.2 (112.1)	—
Dopamine agonists^a^	5.0 (45.5%)	—
Clinical measures		
Retropulsion test	1.6 (1.4)	1.5 (1.4)
Comfortable 6 m walk test (s)	5.9 (2.4)	6.3 (3.3)
Quick 6 m walk test (s)	4.1 (1.0)	4.1 (0.9)
Timed up and go (s)	18.9 (5.7)	17.4 (3.4)
DBS information		
Time since STN-DBS (years)	4.1 (2.3)	—
Euclidean distance^b^	2.36 (0.32 to 5.17)	—
X distance (negative = medial)^b^	−0.90 (−3.17 to 1.72)	—
Y distance (negative = posterior)^b^	−0.35 (−1.80 to 3.45)	—
Z distance (negative = inferior)^b^	−0.74 (−4.33 to 2.68)	—
Frequency (Hz)	127.2 (14.9)	60.0 (0.0)
Amplitude (V)	3.3 (0.6)	4.7 (1.1)
Pulse width (μs)	62.5 (5.5)	62.5 (5.5)

Abbreviations: ABC-6, 6-item Activities-specific Balance Confidence scale; HFS, high-frequency stimulation; LFS, low-frequency stimulation; PDQ-8, 8-item Parkinson’s Disease Questionnaire; MDS-PDRS-III, Motor subscale of the Movement Disorders Society-Sponsored Revision of the Unified Parkinson’s Disease Rating Scale.

All data represent mean (±1 standard deviation) unless absolute numbers (percentage of sample) marked with^a^, or mean (range) marked with^b^.

### Standing Balance

For this project, measures of balance were derived from the COP data using the BioAnalysis software (Advanced Mechanical Technology, Watertown MA, USA). Specifically, these measures included the range of both the anterior–posterior (AP; distance between the most anterior and posterior COP positions) and medial–lateral (ML; distance between the left- and right-most positions of the COP trajectory), the variability of both the AP and ML sway patterns (as determined using the standard deviation), 95% elliptical sway area (cm^2^), and sway velocity (cm/s). These measures have been used previously to evaluate the efficacy of non-invasive interventions in people with PD^25^ and have been shown to worsen with increased disease severity.^26^ Additionally, the sample entropy measure was used to determine the regularity of the sway patterns in both the AP and ML directions separately.^27^ This procedure used the instantaneous velocity of the AP and ML COP data and expressed the regularity of the time-series on a scale from 0 to 2 (increased values corresponded with less regular sway patterns). For the calculation of sample entropy, the input parameters of *m* = 2 and *r* = .30 were used, in accordance with previous research.^27^

### Gait Initiation

Measures of the anticipatory postural adjustments that precede gait initiation were derived from the COP data collected during the moments preceding the participants’ comfortable walking trials. To facilitate these analyses, the COP data were divided into 2 phases. The first was the postural phase, which included all data between the start of the trial and the point at which the COP reached its maximum posterior and lateral displacement. The second was the locomotion phase, which included all data from the end of the postural phase through to the point where the trailing leg was no longer in contact with the force plate. During each of these phases, outcome measures included the AP sway path length (distance between the most anterior and posterior COP positions), ML sway path length (distance between the left- and right-most COP positions), and average velocity (length of the COP trajectory divided by the trial duration in seconds). The calculation of these outcomes for both the postural and locomotion phases was completed using a custom programme developed in Microsoft Excel.

### Statistical Analysis

Demographic data were reported as aggregate means and standard deviations for the entire group. Primary outcome measures were COP-derived indices of balance during standing (anterior–posterior and medial–lateral range, variability, sway area, sway velocity, anterior–posterior sample entropy, and medial–lateral sample entropy) and gait initiation (anterior–posterior and medial–lateral range, sway area, and sway velocity during both the postural and locomotion phases). To examine differences between high- and low-frequency stimulation conditions, linear mixed model (LMM) analyses with a repeated factor of stimulation (2 levels) were used. Six additional models were then run in which the following variables were entered individually as covariates; the Euclidean distance; *X* distance; *Y* distance; *Z* distance; the TEED, and the MDS-UPDRS III. To provide insight into the clinical meaningfulness of any changes in static balance, and/or gait initiation, the minimal detectable change (MDC) for each measure was also derived. The MDC represents the minimum change in a particular outcome measure that would be considered to represent a meaningful change in patient function and, hence, provides useful information regarding the clinical importance of the reported findings. All statistical procedures were conducted using the Statistical Package for the Social Sciences (Version 22, SPSS Inc., USA), with the estimated marginal means and standard errors considered against a *P* < .05 level of significance. Following completion of all data analyses, the 2 therapeutic conditions were re-identified to allow appropriate interpretation and discussion of the outcomes.

## Results

### Study Population

**Figure 1.**
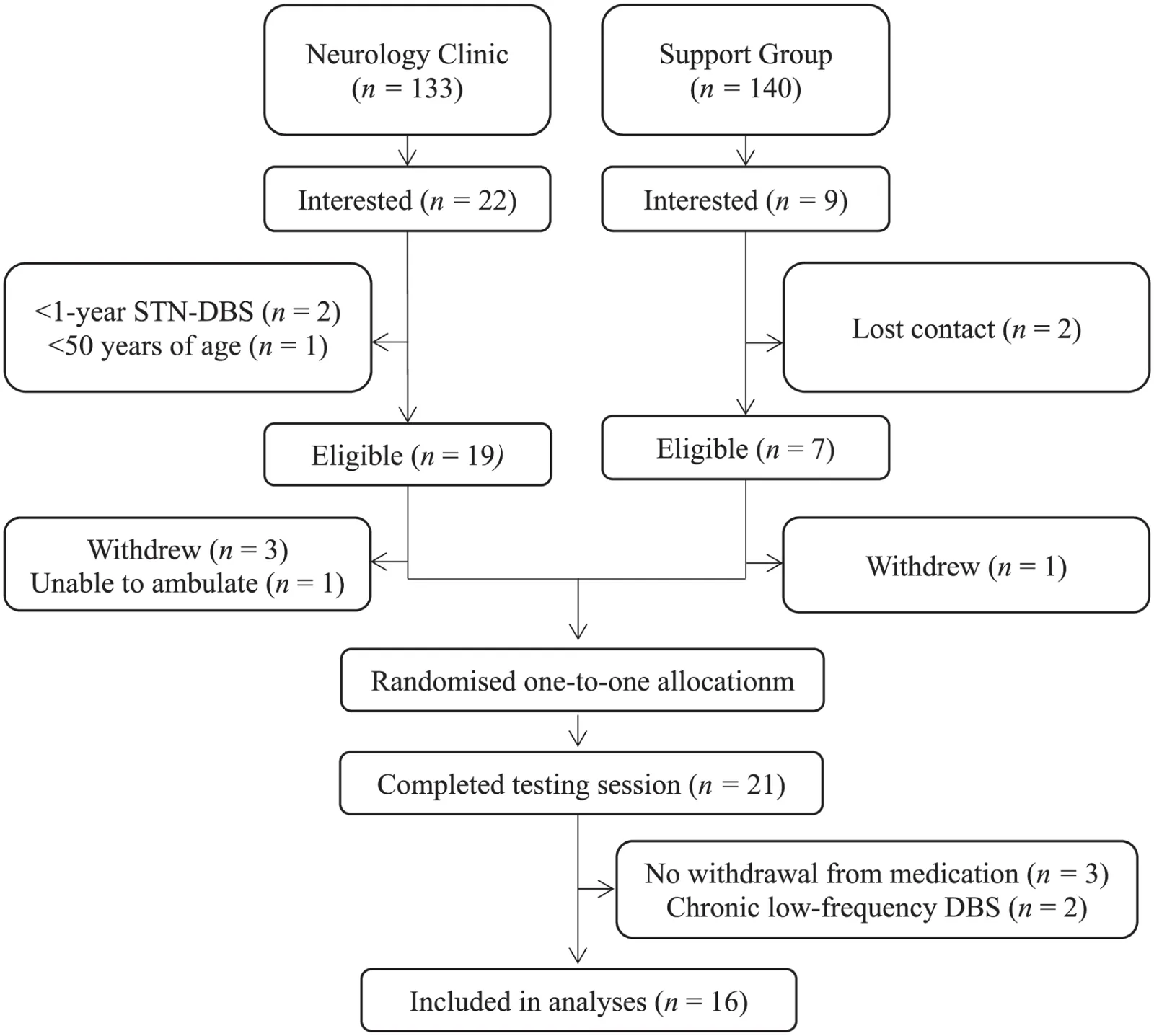
CONSORT diagram summarising the recruitment and screening procedures for those invited to participate.

Between March and August 2018, 31 post-operative STN-DBS PD patients expressed interest to participate in the study. Of these patients, 26 were deemed to be eligible following initial screening and scheduled to attend the data collection session (Figure 1). After eligibility checking, 21 participants completed the study. Following data collection, data for 5 participants were excluded because they took their anti-parkinsonian medication on the morning of testing (n = 3) or because their typical (chronic) stimulation settings already involved low-frequency stimulation (n = 2). All available trial data per condition were included in the LMMs for the remaining 16 participants (Table 1), whose chronic stimulation parameters are reported in Supplemental Table 1. Of the 16 participants, 9 reported experiencing more than 1 fall in the 12 months prior to assessment.

### Standing Balance

LMM analyses returned a significant effect for stimulation (high- vs low-frequency) for sway velocity and ML and AP sample entropy (Table 2). Sway velocity was significantly reduced during low-frequency stimulation and this difference was independent of electrode location (active contacts were located within or immediately adjacent to the STN) and motor symptom severity. Similarly, during the low-frequency stimulation condition, the regularity of AP and ML sway was also reduced (ie, lower sample entropy values representing greater regularity) relative to the high-frequency stimulation state. While the lower values for ML sway regularity were independent of electrode placement, TEED and motor symptom severity, the differences in AP sway regularity were negated after accounting for differences in electrode placement. There were no significant effects of assessment order, with the outcomes collected for those receiving HFS first being comparable (not statistically significantly different) to the outcomes recorded for participants receiving LFS first.

**Table 2.** Force Plate Derived Measures During Standing Postural Stability for the HFS and LFS STN-DBS Conditions.

Measures	HFS	LFS	Effect size [confidence interval]	Minimal detectable change	Covariates			
					None	ED (2.318)	TEED (105.50)	MDS-UPDRSIII (32.69)
Medial–lateral range (cm)	1.75 (0.85)	1.59 (0.59)	0.282 [−0.267, 0.830]	0.020	ns	ns	ns	ns
Anterior–posterior range (cm)	2.99 (1.10)	3.12 (1.09)	−0.113 [−0.918, 0.692]	0.042	ns	ns	ns	ns
Variability of medial–lateral sway (cm)	0.37 (0.23)	0.32 (0.12)	0.062 [−0.072, 0.197]	0.001	ns	ns	ns	ns
Variability of anterior–posterior sway (cm)	0.64 (0.27)	0.64 (0.26)	−0.058 [−0.242, 0.125]	0.002	ns	ns	ns	ns
Sway area (cm^2^)	4.13 (3.38)	3.42 (1.86)	0.759 [-.534, 2.051]	0.251	ns	ns	ns	ns
Sway velocity (cm/s)	4.19 (3.39)	3.77 (0.98)	−0.412 [−0.939, 0.115]	0.289	**0.016**	**0.027**	**0.016**	**0.011**
Medial–lateral sample entropy	1.31 (0.27)	1.18 (0.34)	−0.010 [−0.336, 0.31	0.005	**0.023**	**0.025**	**0.023**	**0.020**
Anterior–posterior sample entropy	0.99 (0.32)	0.78 (0.28)	0.217 [0.062, 0.361]	0.004	**0.010**	ns	**0.011**	**0.020**

Abbreviations: ED, Euclidian distance; HFS, High-frequency stimulation; LFS, Low-frequency stimulation; TEED, Total Electrical Energy Delivered; MDS-PDRS-III, Motor subscale of the Movement Disorders Society-Sponsored Revision of the Unified Parkinson’s Disease Rating Scale; ns, No difference; *X*, *Y*, and *Z*. Difference between the ideal and actual locations of the active electrode in the *X* (negative = more medial), *Y* (negative = more posterior), and *Z* (negative = more inferior) directions.

Data represent means (±1 standard deviation) with *P*-values in bold.

### Gait Initiation

No significant differences were identified between low- and high-frequency for any of the balance measures derived from the postural phase of gait initiation. However, during the locomotion phase of gait initiation, low-frequency stimulation led to increased ML range, sway area, and average velocity compared to high-frequency stimulation. The difference observed for sway area was independent of electrode location, while the changes in both ML range and average velocity appeared to be explained by variations in electrode contact location (Table 3).

**Table 3.** Force Plate-derived Measures for the HFS and LFS STN-DBS Conditions During Gait Initiation.

Measures	HFS	LFS	Effect size [confidence interval]	Minimal detectable change	Covariates			
					None	ED (2.318)	TEED (93.46)	MDS-UPDRS-III (−31.6)
Postural								
Medial–lateral range (cm)	4.49 (10.74)	4.39 (13.23)	0.08 [−0.47, 0.62]	0.478	ns	ns	ns	ns
Anterior–posterior range (cm)	2.2 (9.42)	2.63 (12.72)	−0.33 [−1.08, 0.41]	0.449	ns	ns	ns	ns
Sway area (cm^2^)	4.31 (5.59)	6.05 (9.04)	−0.21 [−0.91, 0.49]	2.93	ns	ns	ns	ns
Average velocity (cm/s)	2.44 (6.49)	3.02 (15.04)	−0.52 [−1.20, 0.15]	0.453	ns	ns	ns	ns
Locomotion								
Medial–lateral range (cm)	1.19 (6.49)	1.59 (8.37)	−0.50 [−0.96, −0.03]	0.306	**0.03**	ns	**0.031**	**0.037**
Anterior–posterior range (cm)	5.58 (14.51)	5.95 (17.32)	−0.34 [−1.11, 0.43]	0.64	ns	ns	ns	ns
Sway area (cm^2^)	3.66 (17.99)	7.04 (40.20)	−1.17 [−1.82, −0.51]	1.36	**<0.001**	**<0.001**	**0.001**	**0.001**
Average velocity (cm/s)	10.99 (33.77)	13.88 (55.56)	−0.65 [−1.25, −0.05]	1.873	**0.03**	ns	**0.042**	**0.034**

Abbreviations: ED, Euclidian distance; HFS, high-frequency stimulation; LFS, low-frequency stimulation; TEED, total electrical energy delivered; MDS-PDRS-III, motor subscale of the Movement Disorders Society-Sponsored Revision of the Unified Parkinson’s Disease Rating Scale; ns, No difference; *X*, *Y*, and *Z*. Difference between the ideal and actual locations of the active electrode in the *X* (negative = more medial), *Y* (negative = more posterior), and *Z* (negative = more inferior) directions.

Data represent means (±1 standard deviation) with *P*-values in bold.

### Symptoms Severity and Clinical Measures

There were no differences in any of the clinical measures of mobility (6-m walk AND Timed Up and Go) or symptom severity (MDS-UPDRS III AND retropulsion test) between the low- and high-frequency stimulation conditions. Whilst 5 participants had reported freezing of gait symptoms affecting their daily lives, no freezing episodes took place during data collection. Of the 16 participants who completed the assessments, 10 experienced worsening symptoms of resting tremor with low-frequency stimulation. Six of these were able to complete the assessments without difficulty, but the remaining 4 were unable to complete the assessments while receiving low-frequency stimulation. Secondary analyses that included only those participants who were able to complete the assessments under both therapeutic conditions confirmed that the reported findings were not biased by the 4 participants who were unable to complete the low-frequency STN-DBS condition. There was no difference in age, disease duration, time since surgery, or electrode location for those who were unable to complete the low-frequency STN-DBS condition.

## Discussion

This study employed a double-blinded randomised crossover design to evaluate the effect of low-frequency STN-DBS on objective measures of stability during standing balance and gait initiation in people with PD. Low-frequency STN-DBS (60 Hz), with a commensurate voltage increase to maintain the TEED of the usual high-frequency DBS (chronic) level, improved postural control during standing and gait initiation in post-operative STN-DBS PD. With respect to clinical measures, there were no differences for the retropulsion test between the high- and low-frequency stimulation conditions. Similar findings have been reported in separate studies that evaluated the efficacy of low-frequency stimulation on largely subjective assessments of balance.^16-18,28,29^ It is possible that clinical measures of balance lack the sensitivity to detect subtle changes evident in people with PD with STN-DBS. A battery of tests, including objective force plate measures of balance might be best suited to assessing balance in this clinical population.^30^

Research investigating the potential benefits of low-frequency STN-DBS for improving balance in people with PD has provided inconsistent findings, with some reporting improvements^14,15^ and others describing no difference.^16-18^ The current study extends on this earlier research by incorporating objective force plate measures and employing a randomised cross-over design to further examine the effects of low-frequency STN-DBS therapy on balance. Our findings are potentially important, as previous research has shown that, despite of its capacity to alleviate medication-induced postural instability,^31^ high-frequency STN-DBS has only a limited capacity for improving symptoms of postural instability in people with PD.^32^ Whilst the exact mechanism of change is not yet certain, LFS may not attenuate oscillatory activity as much as compared to HFS, which^33^ A recent study of multi-network cortical activity and functional connectivity comparing STN-DBS OFF, HFS, and LFS found that LFS enhanced.^34^ Stefani demonstrated.^35,36^ The frequency-dependent effects observed in the current study are likely underpinned by LFS engaging associative STN subregions and their connections to other cortical areas and pedunculopontine nuclei. This pattern is not unexpected, as these circuits play a central role in integrating anticipatory postural adjustments and locomotor control and therefore, facilitating cortico-subthalamic–brainstem communication would be expected to preferentially enhance axial, medial–lateral stability during dynamic tasks such as gait initiation.

To our knowledge, this is the first study to evaluate the regularity of standing balance following STN-DBS using the sample entropy measure. Our results show that low-frequency stimulation resulted in more regular sway patterns (ie, lower sample entropies) than high-frequency stimulation. It has been suggested that more regular sway patterns were indicative of better standing balance, as younger adults exhibited more regular sway than elderly fallers and non-fallers.^37^ However, a more regular sway may be reflective of a postural control system that has reduced flexibility and, hence an impaired capacity to adapt to different conditions. Support for this notion is provided by studies that report more regular sway for community-dwelling older adults who fall compared with non-fallers^38^ and more regular sway for people with PD compared to controls.^39^ Considering these collective findings, it seems that sample entropy may provide unique insight into the impact of disease on balance and/or the effect of different therapies on symptom management.

During gait initiation, low-frequency stimulation had no significant impact on sway measures during the postural phase of gait initiation. However, during the locomotion phase of gait initiation, ML range, sway velocity, and sway area were all increased compared to high-frequency. Given reduced sway area is known to correspond with increased symptom severity in people with PD,^9^ and sway area reduces with high-frequency STN-DBS,^12^ the greater sway area observed with low-frequency stimulation during the locomotion phase may reflect improved gait initiation. Furthermore, the increased sway velocity exhibited by participants with low-frequency STN-DBS during the locomotion phase, was indicative of a more dynamic movement pattern during this transition period between standing balance and steady-state walking.^40^ These findings suggest that low-frequency stimulation might be a useful alternative strategy for improving balance and mobility during gait initiation. This finding is consistent with previous systematic evidence that shows that, compared to high-frequency stimulation, low-frequency STN-DBS improves gait patterns in people with PD.^13^

The consistent finding across standing and gait initiation was that low-frequency STN-DBS predominantly affected medial–lateral sway, with comparatively smaller and less consistent effects in the anterior–posterior direction. Anterior–posterior sway is primarily regulated by an ankle-based stiffness strategy, whereas medial–lateral control relies more heavily on hip abductor–adductor activity and frontal-plane stabilisation of the trunk and pelvis.^41^ Accordingly, if low-frequency STN-DBS preferentially enhances axial and proximal postural control, its effects would be expected to manifest more consistently in medial–lateral stability. Analyses of postural sway have demonstrated that medial–lateral control is more sensitive to changes in task difficulty, somatosensory input, and central integration than anterior–posterior sway, reflecting greater reliance on proximal and axial control strategies.^42^ This interpretation is further supported by the phase-specific findings during gait initiation. While no stimulation-frequency effects were observed during the postural phase, low-frequency STN-DBS was associated with significant changes in medial–lateral sway range and sway area during the locomotion phase, with no corresponding effects in the anterior–posterior direction. Together, these results suggest that low-frequency STN-DBS preferentially modulates frontal-plane, axial control mechanisms that become particularly important during dynamic weight transfer and step initiation.

Given the lack of differences between high- and low-frequency STN-DBS during the postural phase of gait initiation, our findings also suggest that the mechanisms responsible for controlling balance during standing and locomotor tasks (eg, gait) may differ.^43^ Although we are unable to compare these improvements with a pre-surgical state, our results indicate that low-frequency STN-DBS therapy that is administered with a voltage change that serves to maintain the TEED improved gait initiation in people with PD who have STN-DBS. These findings potentially provide evidence for the utility of alternate STN-DBS stimulation parameters for people with PD who continue to experience significant gait impairment following the procedure. It must be noted that this alternate stimulation was not tolerated by all and, for 10 participants, the gait improvements came at the cost of the re-emergence of limb tremor. Of these participants, 6 were able to complete the assessments with low-frequency stimulation, while the remaining 4 were unable to complete the assessments at the alternate frequency. A similar re-emergence of tremor was reported in a separate study evaluating the effects of low-frequency STN-DBS^17^; highlighting the need for individualised programming and careful selection of those likely to benefit. At this time, individuals with less severe tremor may be best placed to receive low-frequency STN-DBS.^44^ It should be noted that participants were off medication during testing, and it is possible that the re-emergence of tremor with low-frequency stimulation could be mitigated with concurrent pharmacological management, such as increased levodopa. This factor should be considered in future studies. Furthermore, future research should continue to investigate the effect of alternate stimulation settings such as high and low frequency dual frequency.^45^

### Limitations

Though the 60-minute wash-in/wash-out period was commensurate with studies that have adopted similar methodologies,^14,24^ it is arguable that a longer wash-in period (ie, more than the 60 minutes used in this study) may have been needed. Nevertheless, the relatively short time between 1 stimulation condition and the other means that the results presented in this paper should be considered to represent the participants’ acute responses to low-frequency stimulation. Longitudinal studies are required to determine the long-term efficacy of low-frequency stimulation for people with PD following STN-DBS. A second limitation relates to the number of statistical comparisons performed. Given the exploratory nature of this study, formal correction for multiple comparisons was not applied. Instead, effect sizes, confidence intervals, and MDC values were reported to support interpretation of the magnitude of change and the clinical relevance of findings. A third potential limitation of this research is that participants were assessed following overnight withdrawal from their anti-parkinsonian medications, meaning that even during high-frequency stimulation condition for 50% of the participants (ie, those who usually took medication) was not reflective of their best therapeutic state. However, by removing the potential influence of anti-parkinsonian medications from our assessments of standing balance and gait initiation, we felt that we could better attribute any changes in outcome to the specific stimulation conditions. These limitations should be considered when interpreting the implications of this study’s outcomes. Future research should address these limitations and consider instrumenting the retropulsion test with a force platform to better characterise the effects of LFS on clinically relevant assessments of balance perturbations to strengthen the evidence for the clinical utility of this stimulation strategy.

## Conclusions

During low-frequency STN-DBS, people with PD exhibited improved objective measures of balance during standing and gait initiation compared to their chronic high-frequency STN-DBS treatment. However, in the absence of anti-parkinsonian medication, low-frequency stimulation was not well tolerated by all participants, as some experienced a re-emergence of resting tremor. Furthermore, low-frequency stimulation resulted in more regular sway patterns, though what this means for both balance and falls risk requires further research.

## Supplemental Material

sj-docx-1-nnr-10.1177_15459683261448483 – Supplemental material for Low-Frequency STN-DBS for Standing and Gait Initiation in Parkinson’s Disease: A Double-Blinded Randomised Cross-Over Feasibility Trial Supplemental material, sj-docx-1-nnr-10.1177_15459683261448483 for Low-Frequency STN-DBS for Standing and Gait Initiation in Parkinson’s Disease: A Double-Blinded Randomised Cross-Over Feasibility Trial by Zachary J. Conway, Peter A. Silburn, Thushara Perera, Karen O’Maley, Liam Johnson and Michael H. Cole in Neurorehabilitation and Neural Repair
