# Supplementary material for: Low-Frequency STN-DBS for Standing and Gait Initiation in Parkinson’s Disease: A Double-Blinded Randomised Cross-Over Feasibility Trial
Source: Neurorehabil Neural Repair. 2026 Jun 4;40(10):874–84. doi: 10.1177/15459683261448483 (PMC13612862; doi:10.1177/15459683261448483)
Supplement: Supplementary file 1 [file sj-docx-1-nnr-10.1177_15459683261448483.docx]

Supplementary Table 1: Chronic stimulation parameters

| **Participant** | **V** | **F** | **PW** |
| --- | --- | --- | --- |
| 1 | 3 | 130 | 75 |
| 2 | 4 | 160 | 70 |
| 3 | 4 | 155 | 75 |
| 4 | 3 | 110 | 60 |
| 5 | 3 | 140 | 60 |
| 6 | 3 | 120 | 60 |
| 7 | 3 | 110 | 60 |
| 8 | 4 | 130 | 60 |
| 9 | 4 | 130 | 60 |
| 10 | 3 | 120 | 60 |
| 11 | 3 | 130 | 60 |
| 12 | 5 | 130 | 60 |
| 13 | 4 | 130 | 60 |
| 14 | 4 | 110 | 60 |
| 15 | 3 | 115 | 60 |
| 16 | 2 | 115 | 60 |
